# Mechanisms of action of naturally occurring antibodies against β-amyloid on microglia

**DOI:** 10.1186/1742-2094-10-5

**Published:** 2013-01-14

**Authors:** Maike Gold, David Mengel, Stephan Röskam, Richard Dodel, Jan-Philipp Bach

**Affiliations:** 1 Department of Neurology, Philipps-University of Marburg, Baldingerstraße 1, 35043, Marburg, Germany

**Keywords:** Alzheimer’s disease, β-amyloid, Microglia, Inflammation, Immunoglobulins

## Abstract

**Background:**

Naturally occurring autoantibodies against amyloid-β (nAbs-Aβ) have been shown to exert beneficial effects on transgenic Alzheimer’s disease (AD) animals *in vivo* and on primary neurons *in vitro.* Not much is known about their effect on microglial cells. Our aim was to investigate the effect of nAbs-Aβ on amyloid-β (Aβ)-treated microglial cells *in vitro* with respect to cell viability, stress pathways, cytokine production and phagocytotic abilities and whether these effects can be conveyed to neurons.

**Methods:**

Primary microglial cells isolated from Swiss Webster mouse mesencephalons on embryonic day 13.5 were pretreated with nAbs-Aβ and then treated with Aβ oligomers. After 3 hours, phagocytosis as well as western blot analysis were evaluated to measure the amount of phagocytized Aβ. Cell viability was analyzed using an MTT assay 24 hours after treatment. Pro-inflammatory cytokines in the supernatants were analyzed with ELISAs and then we treated primary neuronal cells with these conditioned microglia supernatants. Twenty-four hours later we did a MTT assay of the treated neurons. We further investigated the effect of a single nAbs-Aβ administration on Tg2576 mice *in vivo.*

**Results:**

Upon co-administration of Aβ and nAbs-Aβ no change in microglia viability was observed. However, there was an increase in phosphorylated p38 protein level, an increase in the pro-inflammatory cytokines TNF-α and IL-6 and an increase in Aβ uptake by microglial cells. Treatment of primary neurons with conditioned microglia medium led to a 10% improvement in cell viability when nAbs-Aβ were co-administered compared to Aβ-treated cells alone. We were unable to detect changes in cytokine production in brain lysates of Tg2576 mice.

**Conclusions:**

We provide evidence on the mechanism of action of nAbs-Aβ on microglia *in vitro*. Interestingly, our *in vivo* data indicate that nAbs-Aβ administration should be considered as a therapeutic strategy in AD, since there is no inflammatory reaction.

## Background

Alzheimer’s disease (AD) is a neurodegenerative disorder leading to the loss of cholinergic neurons in the brain, characterized by extracellular amyloid-β (Aβ) deposits and the formation of neurofibrillar tangles comprising hyperphosphorylated tau protein inside the cell. The toxicity of Aβ deposits is conveyed by oligomeric aggregates that lead to synaptic dysfunction and apoptosis of neurons [1,2].

Active immunization strategies using different forms of Aβ have been extensively tested in amyloid precursor protein (APP) transgenic mouse models. The initial animal results were promising [3,4], but since active immunization in AD patients was associated with considerable side effects [5] and some patients did not develop Aβ-antibody titers [6], passive immunization strategies came into focus. Treatment with humanized monoclonal antibodies [7] as well as passive immunization with intravenous immunoglobulins (IVIg) has been investigated in pilot clinical trials [8,9]. The rationale for its use is that IVIg contain naturally occurring autoantibodies against Aβ (nAbs-Aβ). Naturally occurring autoantibodies in the central nervous system are involved in maintaining homeostasis by removing debris and are known to prevent inflammation [10]. The beneficial effects of nAbs-Aβ have been shown both *in vitro* on primary neurons and neuronal cell lines as well as *in vivo* in transgenic mice [11,12]. The effect of IVIg on microglial cells has already been investigated by other groups. It has been shown that IVIg reduce phagocytosis *in vitro* via Fc receptors [13], IVIg induce tumor necrosis factor-α (TNF-α) and nitric oxide (NO) in a dose-dependent manner, whereas the greater the IgM/IgA content the higher the impact on microglial cells [14], and that IVIg enhance the secretion of matrix metalloproteinase 9, which seems to play a role in the pathogenesis of multiple sclerosis [15]. Referring to Aβ, Magga *et al*. found an increase in Aβ clearance when they administered IVIg in a dose-dependent manner on primary microglial cells [16]. IVIg depleted of nAbs-Aβ did not promote clearance, indicating that the enhanced clearance effect is mediated by nAbs-Aβ. In a study on the immortalized murine microglial cell line BV-2, administration of IVIg restored cells’ viability and enhanced phagocytotic ability of fibrillar Aβ [17]. In addition to these results, IVIg are limited in their availability and we therefore decided to use nAbs-Aβ in our study. Theoretically, these will be able to be cloned eventually and therefore the problem of availability can be overcome. In all experiments we used the negative fraction of IVIg, which contains all antibodies except those that were purified with the affinity chromatography, and we were not able to see the same effects as those obtained with nAbs-Aβ, coming to the conclusion that nAbs-Aβ are the active substance in IVIg responsible for the beneficial effects with regard to AD. The effect and the interaction, however, of nAbs-Aβ on microglial cells have not been investigated to date. This is especially important as microglia are purported to have a major role in the pathogenesis and propagation of AD [18]. Upon stimulation, microglial cells can react in many different ways, including phagocytosis, the secretion of immunomodulating cytokines and finally programmed cell death in order to kill pathogens or restore tissue integrity [19]. Activated microglial cells have been found surrounding plaques in histopathological sections of AD patients [20] and their role in the phagocytosis of all forms of Aβ has been investigated. Due to the immune-stimulating effect of foreign monoclonal antibodies in the human body, we were interested in the effect of nAbs-Aβ on primary microglial cells. In the following experiments we investigated the effect of nAbs-Aβ on Aβ-treated microglial cells with respect to cell viability, neuroinflammation and phagocytosis, and whether any effect on primary neurons is conveyed by treated microglial cells.

## Material and methods

All chemicals were obtained from Sigma-Aldrich, St. Louis, MO, USA unless indicated otherwise.

### Antibodies

Antibodies against the following proteins were used: Aβ (clone 6E10, Covance, Princeton, NJ, USA), phosphorylated (phospho) p38, horseradish peroxidase (HRP)-conjugated secondary antibodies (all Cell Signaling Technology, Danvers, MA, USA), glyceraldehyde 3-phosphate dehydrogenase (GAPDH) (Novus Biologicals, Littleton, CO, USA), allophycocyanin (APC)-conjugated CD11b (eBioscience, San Diego, CA, USA).

### Cells

#### Primary microglial cell culture

For the isolation of primary microglial cells we used a protocol based on that described by Saura *et al*. [21]. Microglial cells were derived from embryonic day 13.5 (E13.5) Swiss Webster mouse mesencephalons. Briefly, meninges-free mesencephalons were isolated, collected and homogenized in Leibovitz L-15 medium (PAA Laboratories, Pasching, Austria). After centrifugation cells were resuspended in Dulbecco's modified Eagle’s medium (DMEM) with L-glutamine (Lonza, Basel, Switzerland) containing 10% fetal bovine serum (FBS) (PAA Laboratories, Pasching, Austria), 100 U/mL penicillin and 100 μg/mL streptomycin (Lonza, Basel, Switzerland) and plated on polyethyleneimine (PEI)-coated plates. After 7 days cells were stimulated with granulocyte-macrophage colony-stimulating factor (GM-CSF) (Roche, Basel, Switzerland). Cells were subplated on PEI-coated plates and used for experiments on day *in vitro* 14 to 19.

#### Primary cortical neuron cell culture

Neurons were cultured from cortices of E13.5 Swiss Webster mice. Briefly, meninges-free cortices were isolated, collected and homogenized in Leibovitz L-15 medium and resuspended in Neurobasal-A Medium (Invitrogen, Grand Island, NY, USA) supplemented with B27 (Gibco, Basel, Switzerland), 100 U/mL penicillin and 100 μg/mL streptomycin and L-glutamine and plated on PEI-coated plates. Cells were used for experiments on day 6 to 8.

### Animals

Twenty- to 22-month-old heterozygous adult female Tg2576 mice expressing mutant APP_SWE (695(K670N,M671L)_ under the control of the hamster prion promoter in a hybrid C57Bl/6 × SJL background and age- and gender-matched non-transgenic wild-type control mice (WT) were used for all experiments. Tg2576 and WT mice were randomly divided into groups of five to six, independent of genotype and treatment, on a 12 hour light–dark schedule (lights on 07:00 to 19:00). They had free access to tap water, were fed *ad libitum* and kept under standard conditions. The sample sizes of the groups were as follows: transgenic (Tg) control n = 5, Tg nAbs-Aβ n = 5, WT n = 6. All animal procedures were approved by the office of the district president and the Institutional Animal Care and Use Committee.

### Administration of nAbs-Aβ to mice

Mice were treated intraperitoneally (i.p.) with nAbs-Aβ (400 μg dissolved in 0.2 ml of physiological saline solution) or vehicle (0.2 ml physiological saline solution)*.* The mice were sacrificed 24 hours later and samples were taken. Brains were immediately frozen in liquid nitrogen and stored at −80°C.

### Preparation of oligomeric Aβ

Aβ oligomers were synthesized according to Kayed *et al*. [22]. Briefly, lyophilized synthetic Aβ_42_ peptide (PSL, Heidelberg, Germany) was initially monomerized by dissolving in hexafluoroisopropanol (HFIP) and separated into aliquots in low-binding tubes. HFIP was evaporated and aliquots were stored at −20°C until use. The peptide film was resuspended in ultrapure water with a final concentration of 232 μM. A small magnetic stirrer was added and the peptide solution was stirred for 48 hours at room temperature at 1400 rpm.

### Preparation of nAbs-Aβ

nAbs-Aβ were isolated from IVIg as previously described [11]. Briefly, we used purified intravenous IgG (Octagam 5%). Ninety-six percent of protein represents normal human IgG (IgA <0.2 mg; IgM <0.1 mg). IgG subclasses are fully represented (IgG 1, 65%; IgG 2, 30%; IgG 3, 3%; IgG 4, 2%). To ensure homogenous orientation of the Aβ peptide to the affinity column, a cysteine residue was introduced at the N-terminal part of the peptide. The azlactone-activated support contains an iodoacetyl group (Ultralink; Perbio, Bonn, Germany) at the end of a hexadecyl-spacer group, which reacted with the cysteinyl-sulfhydryl group to yield a stable thioether linkage in order to reduce steric hindrance and provide maximum binding capacity of the antibodies. The bound antibodies were eluted from the column with 10 × 0.5 ml 0.1 M glycine buffer, pH 2.8. Each fraction was collected in a microreaction tube containing 35 μl 1 M Tris–HCl, pH 9. IVIg depleted of nAbs, termed flow-through (ft), was also collected and used as a negative control in experimental settings. To maintain the integrity of the antibodies, a neutral pH was adjusted immediately after elution by adding the appropriate amount of Tris-HCI or glycine buffer. nAbs-Aβ and ft were sterile filtered and stored at −20°C until use.

### Cell viability assessment

Quantification of cell viability was performed using the 3-(4,5-dimethylthiazol-2-yl)-2,5-diphenyltetrazolium bromide (MTT) reduction assay. Microglial cells were treated with oligomeric Aβ_42_ (5 μM) and/or nAbs-Aβ or ft (both 0.1 μM and 1 μM) for 24 hours in DMEM without FBS. Medium was changed to medium containing 0.5 mg/mL MTT and incubated for 1 hour. Medium was removed and cells were solubilized with dimethyl sulfoxide (DMSO) (AppliChem, Darmstadt, Germany) and shaken for 30 minutes. The absorbance was measured at 570 nm on a plate reader. Neuronal cells were treated with supernatants of microglial cells. Neurobasal-A medium was removed and conditioned medium from microglial cells was added. After 24 hours, medium was replaced with DMEM containing 0.5 mg/mL MTT and incubated for 1 hour and the assay was performed as described above.

### Measurement of TNF-α, IL-1β, IL-6 and interferon-γ

Primary microglial cells were pre-treated with nAbs-Aβ or ft (0.1 μM) for 30 minutes and stimulated with oligomeric Aβ_42_ (5 μM) for 24 hours. Supernatants were stored at −20°C until use. Mice brains were lysed using T-PER lysis buffer (Pierce, Rockford, IL, USA) containing protease inhibitors (Roche Diagnostics, Mannheim, Germany). Protein concentrations were determined using a spectrophotometer. Concentrations of TNF-α, interleukin-1β (IL-1β), IL-6 and interferon-γ (IFN-γ) were quantified using the Duoset enzyme-linked immunosorbent assay (ELISA) system (R&D, Minneapolis, MN, USA) according to the manufacturer’s protocol.

### Western blot

Treated cells were washed twice with ice-cold phosphate-buffered saline (PBS) and cells were lysed using M-Per supplemented with protease and phosphatase inhibitors according to the manufacturer’s protocol. The protein concentration of the cell lysates was determined using NanoDrop. Total cell protein was separated by 4 to 12% sodium dodecyl sulphate-polyacrylamide gel electrophoresis (SDS-PAGE) and transferred onto nitrocellulose membranes. Membranes were blocked with 1 × Rotiblock (Carl Roth, Karlsruhe, Germany) and incubated with the appropriate primary antibody dilutions for Aβ (1:2000), phospho p38 (1:4000) or GAPDH (1:5000). Membranes were washed with Tris-buffered saline containing 5% Tween20 and incubated with secondary antibodies. Membranes were incubated with SuperSignal™ West Dura Extended Duration Substrate (Thermo Scientific, Rockford, IL, USA) and exposed to an autoradiographic film (CL-Xposure Film, Thermo Scientific, Rockford, IL, USA). Band intensity was quantified using a densitometer.

### Aβ phagocytosis assay

Fluorescein isothiocyanate (FITC)-labeled Aβ_42_ was oligomerized as described above. Primary microglial cells were pre-incubated with nAbs-Aβ or ft (0.1 μM) in serum-free DMEM for 30 minutes at 37°C and then treated with FITC-Aβ_42_ (5 μM) for 3 hours. For flow cytometric analysis, microglial cells were rinsed twice with ice-cold PBS, harvested, and washed with fluorescence-activated cell sorting (FACS) buffer (PBS with 0.1% FBS). Microglial cells were stained with APC-conjugated CD11b antibody, washed and resuspended in FACS buffer containing HOECHST 33258 to sort vital cells only. Measurements were performed with a LSR II flow cytometer (Becton Dickinson, Franklin Lakes, NJ, USA). Analysis was performed using FlowJo software (Tree Star Inc., Ashland, OR, USA). Only vital and CD11b^+^ cells were used for uptake comparison. Uptake of the differently treated cells was evaluated by comparing the mean fluorescence intensity of FITC. Levels of Aβ phagocytosis were also determined by Western blot.

### Statistical analysis

All results of *in vitro* as well as *in vivo* experiments are presented as the mean ± SD. We used the Student’s *t* test to assess the statistical significance of all experiments. For all statistical comparisons, the following definitions were used: *P* <0.05 (*), *P* <0.01 (**) or *P* <0.001 (***).

## Results

### nAbs-Aβ are not toxic to microglia

**Figure 1:**
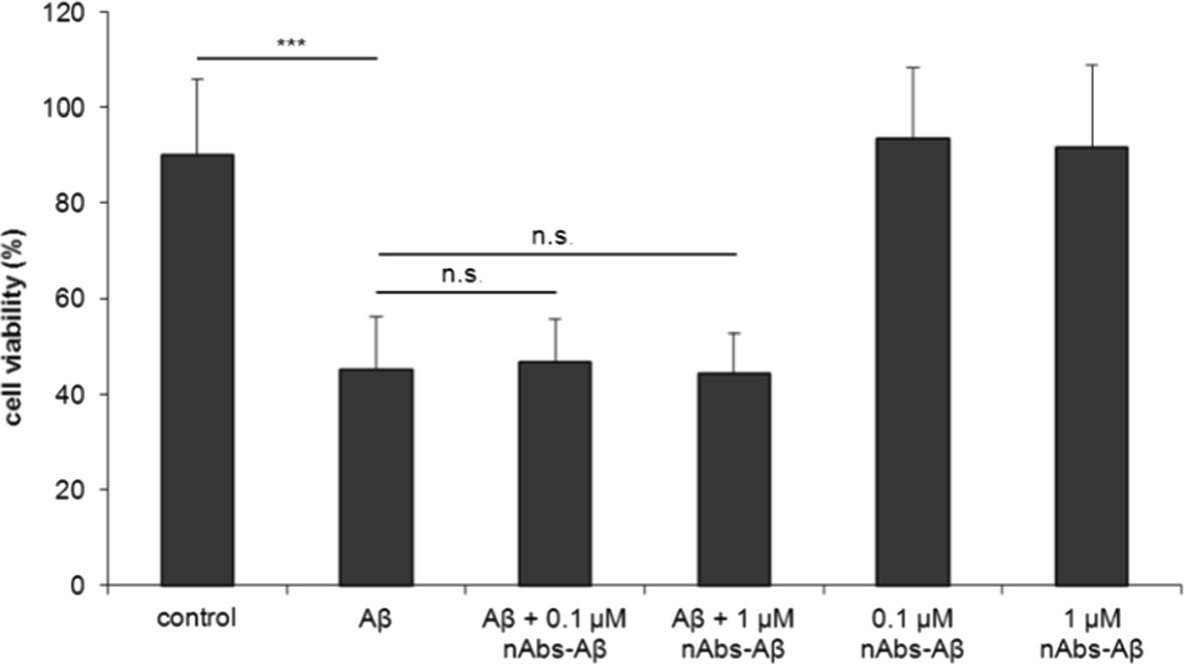
**Aβ oligomers reduce cell viability, co-administered nAbs-Aβ do not attenuate cell death.** Primary microglia cells were treated with Aβ_42_ oligomers, cell viability was measured using the MTT assay. To investigate the effect of nAbs-Aβ, microglial cells were pre-incubated for 30 minutes with different concentrations of nAbs-Aβ. Viability of untreated cells was referred to as 100% viability. Experiments were performed at least three times, independently. nAbs-Aβ, naturally occurring autoantibodies against amyloid-β; MTT, 3-(4,5-dimethylthiazol-2-yl)-2,5-diphenyltetrazolium bromide.

nAbs-Aβ have been shown to have beneficial effects on Aβ oligomer-induced toxicity in neuronal cells [11,12]. It has recently been reported that Aβ oligomers reduce viable microglial cells *in vitro*[23]. We investigated the effect of nAbs-Aβ on Aβ oligomer-induced toxicity (Figure 1). Cell viability was reduced to 50% when stimulated with Aβ_42_ oligomers. nAbs-Aβ had no beneficial effect on the Aβ-induced reduction in viability. nAbs-Aβ alone showed no reduction in cell viability, nor did solvent-treated cells.

### Effect of Aβ oligomers and co-administered nAbs-Aβ on stress pathways and cytokine levels *in vitro*

**Figure 2:**
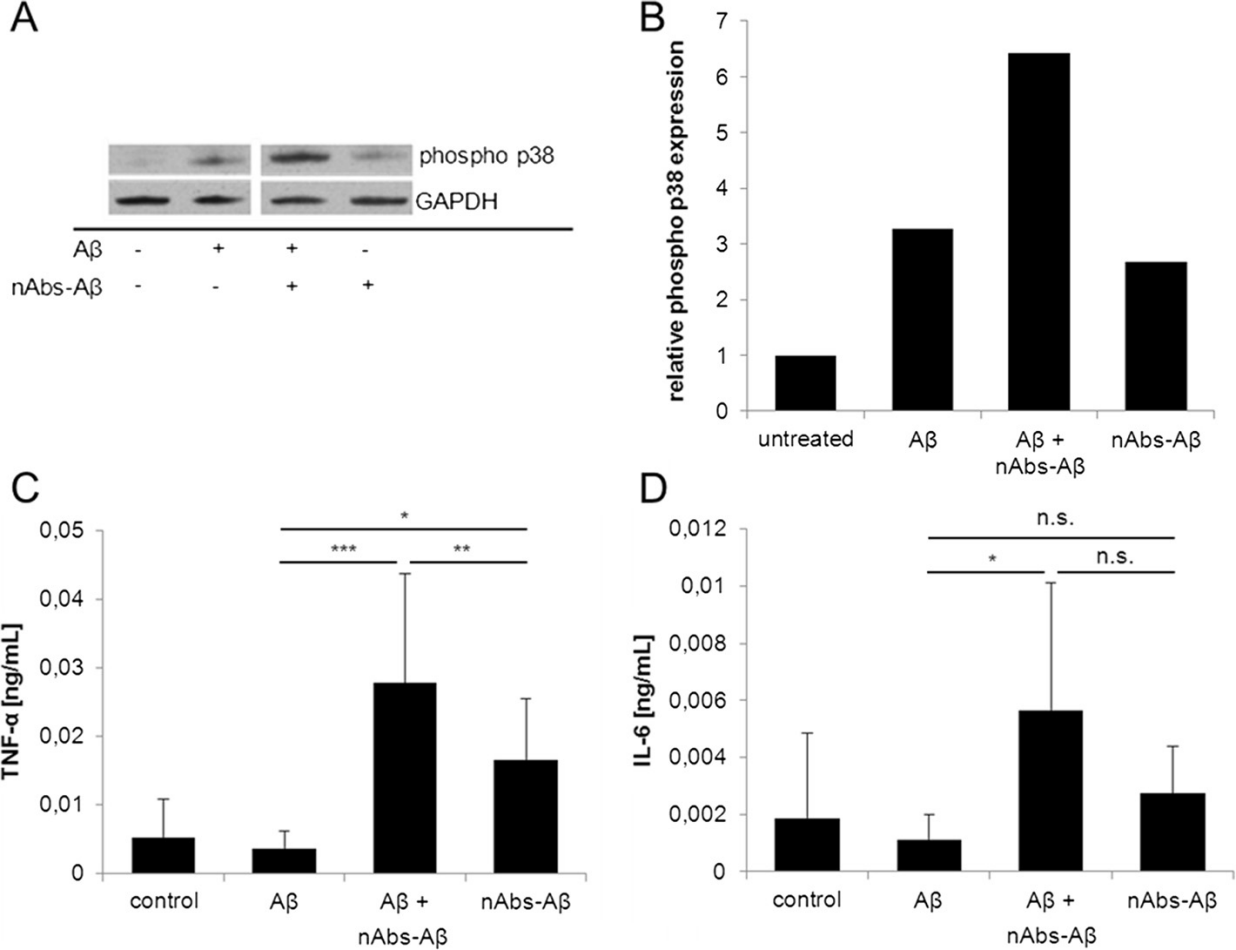
**Co-administration of nAbs-Aβ and Aβ oligomers leads to a rise in phosphorylated p38 and TNF-α levels.** Western blot analysis was performed to detect protein levels of phosphorylated p38. GAPDH was used as loading control **(A)**. Phospho p38 levels were evaluated densitometrically and normalized to GAPDH **(B)**. **C** + **D** show cytokine levels of Aβ-treated cells. Cells were treated for 24 hours and supernatants were subjected to cytokine ELISA. ELISA experiments were performed at least three times, independently. GAPDH, glyceraldehyde 3-phosphate dehydrogenase; ELISA, enzyme-linked immunosorbent assay; nAbs-Aβ, naturally occurring autoantibodies against amyloid-β; TNF-α, tumor necrosis factor-α.

Since Aβ-mediated cell viability was unaffected by nAbs-Aβ, we investigated changes in intracellular stress pathways upon stimulation with Aβ oligomers and in the presence of nAbs-Aβ. We observed an increase in phospho p38 protein levels upon stimulation with Aβ oligomers alone compared to untreated microglial cells (Figure 2A). nAbs-Aβ alone also induced a slight increase in phospho p38 levels. However, co-administration of Aβ oligomers and nAbs-Aβ led to a strong induction of phospho p38 with protein levels increasing 10-fold compared to untreated cells (Figure 2B).

Cytokine levels were examined after 24 hours co-treatment. The results are consistent with the results obtained from the phospho p38 stress-pathway investigation: Aβ and nAbs-Aβ alone slightly increased TNF-α (Figure 2C) and IL-6 (Figure 2D) levels, whereas co-administration of both Aβ and nAbs-Aβ led to a strong and significant induction in pro-inflammatory cytokine levels. Co-administration of ft did not lead to the induction of cytokine levels (data not shown).

### Influence of nAbs-Aβ on pro-inflammatory cytokines in Tg2576 transgenic animals

**Figure 3:**
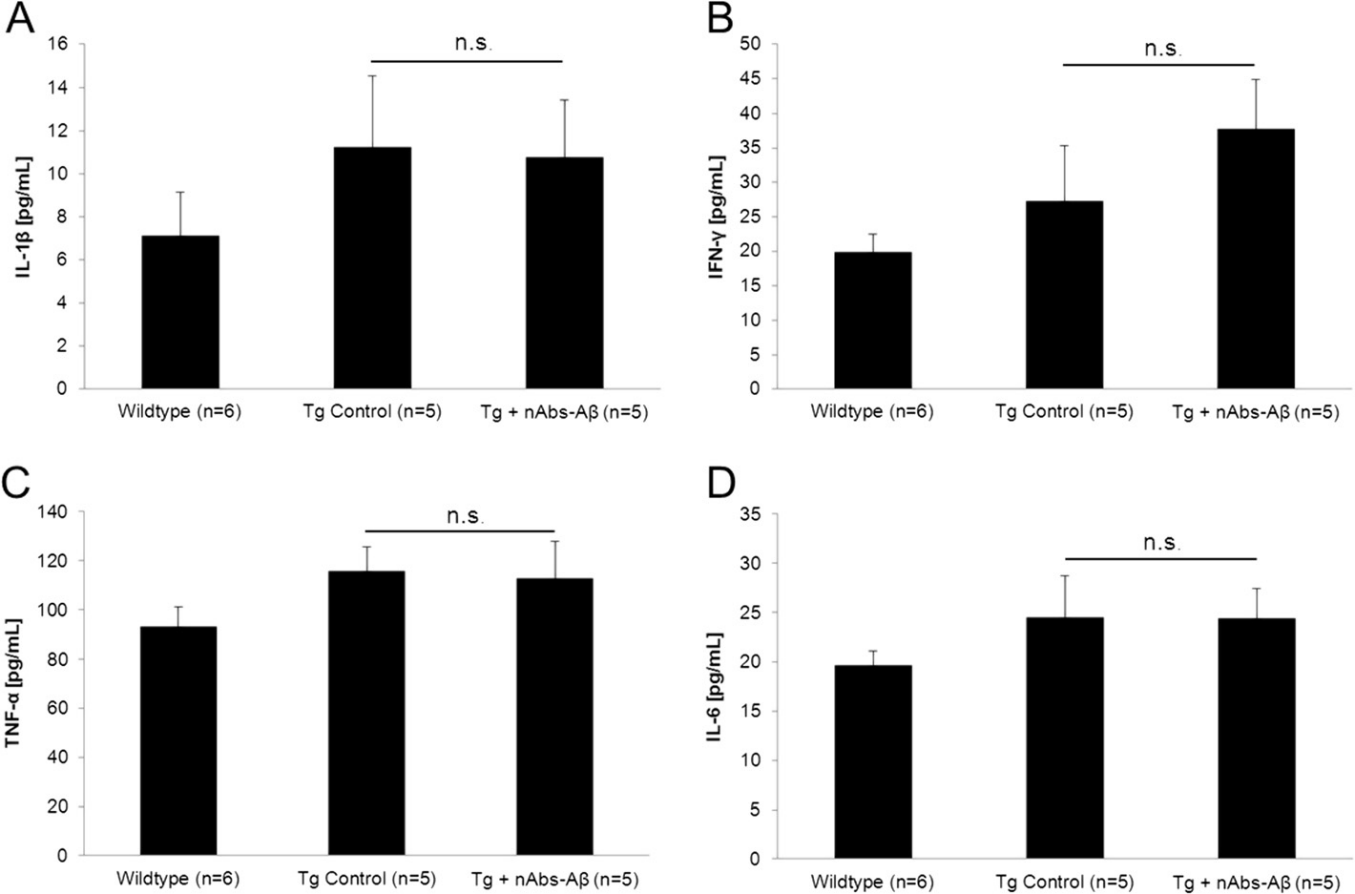
**Injection of nAbs-Aβ does not change cytokine levels in Tg2576 mice brain lysates.** Brain lysates of wild-type mice, Tg2576 mice and Tg2576 mice treated with nAbs-Aβ for 24 hours were subjected to cytokine ELISA. Figure 3 shows cytokine levels for IL-1β **(A)**, INF-γ **(B)**, TNF-α **(C)** and IL-6 **(D)**. ELISA, enzyme-linked immunosorbent assay; IL, interleukin; INF, interferon; nAbs-Aβ, naturally occurring autoantibodies against amyloid-β; TNF-α, tumor necrosis factor-α.

After observing the induction of pro-inflammatory cytokines *in vitro* following treatment of microglial cells with nAbs-Aβ, we next evaluated cytokine concentration in brain homogenates of wild-type animals as well as Tg2576 mice. For all analyzed cytokines we were able to detect upregulation in transgenic animals (Figure 3). IL-1β was elevated by 58% compared to wild-type animals (Figure 3A) (7.11 ± 2.05 vs. 11.23 ± 3.32, *P* <0.05), IFN-γ levels (Figure 3B) in Tg2576 mice brain were increased by 41% (19.78 ± 1.48 vs. 27.91 ± 8.00, *P* <0.05), TNF-α (Figure 3C) was increased by 24% (93.16 ± 7.97 vs. 115.62 ± 9.95, *P* <0.05) and IL-6 (Figure 3D) showed an increase of 24% (19.65 ± 1.48 vs. 24.45 ± 4.32, *P* <0.05), respectively. Treatment of Tg2576 mice with nAbs-Aβ did not lead to a significant induction or attenuation in the brain concentration of any of the aforementioned cytokines.

### nAbs-Aβ increase Aβ_42_ uptake in primary microglial cells

**Figure 4:**
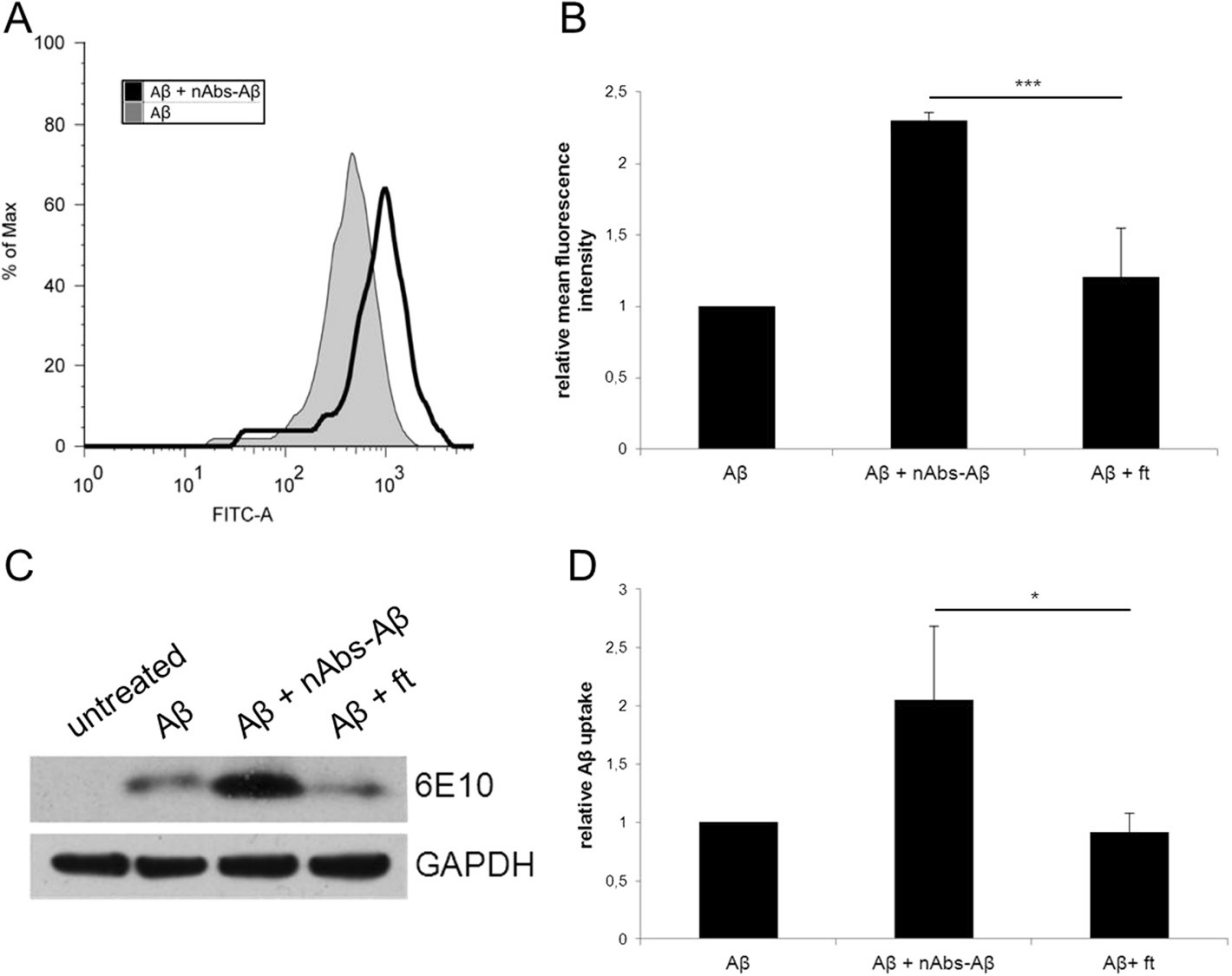
**nAbs-Aβ enhance phagocytosis of Aβ oligomers in primary microglia.** Histogram of the mean fluorescence intensity of FITC-Aβ and FITC-Aβ and nAbs-Aβ (0.1 μM) co-treated cells **(A)**. **(B)** shows the means of three independent experiments. Values were normalized to FITC-Aβ_42_-treated cells. Cell lysates of Aβ_42_-treated cells were also subjected to western blot. To detect Aβ uptake, blots were probed with the monoclonal Aβ antibody 6E10. GAPDH was used as loading control **(C)**. **(D)** shows the densitometric evaluation of three independent experiments; values are given normalized to GAPDH intensity. FITC, fluorescein isothiocyanate; GAPDH, glyceraldehyde 3-phosphate dehydrogenase; nAbs-Aβ, naturally occurring autoantibodies against amyloid-β.

Aβ is taken up by microglial cells from the periphery and it has been shown that the monoclonal antibody 6E10 increases the phagocytotic ability of microglial cells [24]. To investigate the effect of co-administration of Aβ oligomers and nAbs-Aβ on the phagocytotic abilities of microglial cells *in vitro*, we used FITC-labeled Aβ_42_ and FACS analysis. Figure 4A shows a histogram of the shift in the mean fluorescence intensity of the cell population with nAbs-Aβ pre-treatment. Co-administration of nAbs-Aβ led to a doubling in the phagocytosis of Aβ_42_ oligomers (Figure 4B). ft did not enhance phagocytosis, pointing to a genuine effect of nAbs-Aβ.

Levels of phagocytosed Aβ were also determined by western blotting (Figure 4C). The intensity of the protein bands was evaluated densitometrically (Figure 4D). As shown in Figure 4C and D the incubation of primary microglial cells with Aβ and nAbs-Aβ led to a doubling in phagocytosis. In contrast, no significant increase was observed when using ft.

### Supernatants of nAbs-Aβ-co-treated microglial cells have beneficial effects on the viability of neurons compared to supernatants of Aβ-treated microglial cells

**Figure 5:**
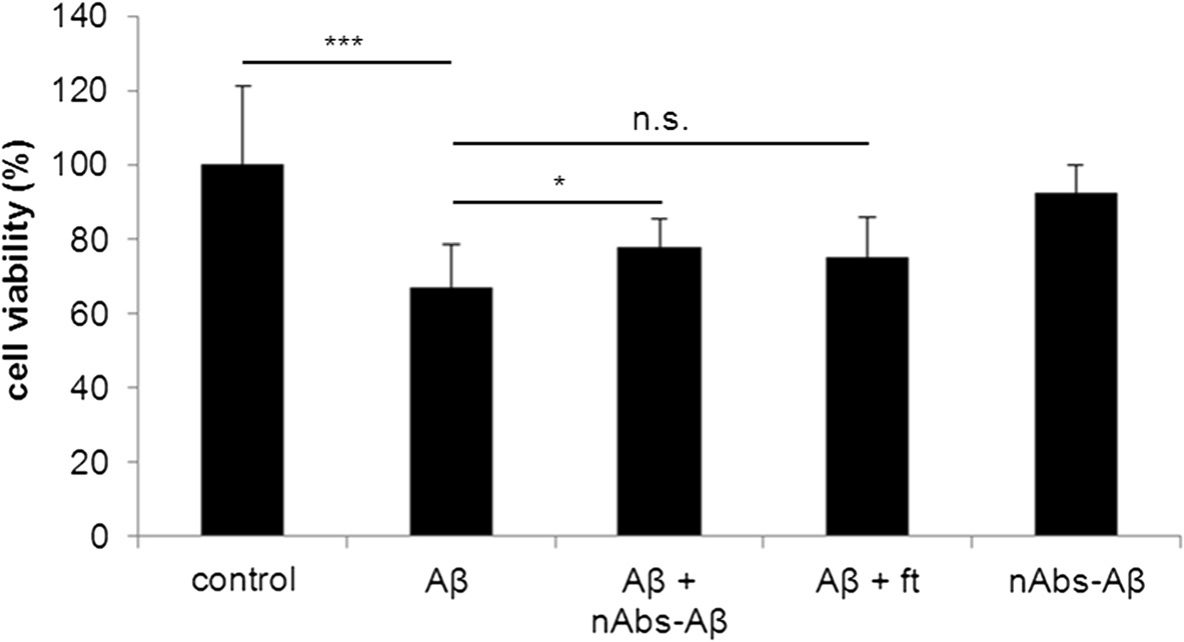
**Beneficial effects of supernatants of nAbs-Aβ co-treated microglial cells on the viability of primary neurons.** Supernatants of treated microglial cells were subjected to primary neurons and incubated for 24 hours. Aβ concentration was 5 μM and nAbs/ft concentration was 0.1 μM. Viability was assessed using MTT assay. Viability of control-treated cells was referred to as 100% viability. Figure 5 shows the means of at least three independent experiments. nAbs-Aβ, naturally occurring autoantibodies against amyloid-β; MTT, 3-(4,5-dimethylthiazol-2-yl)-2,5-diphenyltetrazolium bromide.

To investigate a possible indirect effect of nAbs-Aβ on primary neuronal cells, we cultured neurons in the medium of microglial cells treated with Aβ and/or nAbs-Aβ or ft for 24 hours and assessed cell viability using the MTT assay (Figure 5). Supernatants of microglial cells from three independent preparations were applied to neurons from three independent preparations. Compared to supernatants from solvent-treated microglial cells, supernatants from Aβ-treated microglial cells reduced the viability of neuronal cells to 67%. Following the co-administration of nAbs-Aβ to microglial cells, supernatants significantly reduced the number of viable neuronal cells to 77%. Pre-treatment with ft was not able to significantly restore the viability of primary neuronal cells. In addition, treatment with nAbs-Aβ alone was not harmful to neuronal cells.

## Discussion

The positive effect of nAbs-Aβ on neuronal cells and the fact that IVIg may exert beneficial effects on cognition in AD patients has previously been established [9]. In addition AD patients have reduced levels of nAbs-Aβ in the CSF and plasma compared to age-matched controls, leading to the hypothesis that AD patients will have increased Aβ plaque deposits [25]. In this study we investigated the effect of nAbs-Aβ, isolated from IVIg, on Aβ-exposed microglial cells with the emphasis on cytokine production and phagocytosis of Aβ and whether changes in these physiological actions can convey beneficial effects to primary neurons.

Although the viability of neurons improves with the co-administration of nAbs-Aβ in Aβ oligomer-treated cells [11,12], we found no similar effects on the viability of microglial cells. nAbs-Aβ alone did not influence cell viability, an important property of nAbs-Aβ considering their therapeutic use. Smith *et al*. provided evidence for a link between increased phagocytosis of Aβ and induction of apoptosis in BV-2 microglial cells [26]. This may also hold true for the co-administration of nAbs-Aβ and could explain the difference in the effect observed for neuronal cells. Compared to the monoclonal antibody 6E10, nAbs-Aβ increased pro-inflammatory TNF-α levels *in vitro*, whereas no significant changes were observed for IL-6. With the additional administration of Aβ oligomers TNF-α levels and IL-6 levels rose significantly. Consistent with these results, we observed a strong increase in the phosphorylation of the stress pathway protein MAPK p38. There is contradictory evidence regarding whether pro-inflammatory cytokines are beneficial or harmful in AD; current data support the hypothesis that it is a matter of the right equilibrium [27]. Small rises in pro-inflammatory cytokines seem to have beneficial effects, whereas the induction of excessive and prolonged secretion of pro-inflammatory cytokines can lead to chronic neuroinflammation and neurodegeneration. Neuroprotective effects, especially of TNF-α, have been reported. The neuroprotective effect seems to be mediated by the regulation of peroxide formation, calcium accumulation and NF-κB activation [28]. It has been shown by several groups that Aβ overexpression induces neuroinflammation in the AD transgenic mouse models Tg2576 and B6.Cg-Tg(APPswe, PSEN1dE9)85Dbo/J [29,30]. In our present study, we also observed increased cytokine concentrations in 22-month-old Tg2576 mice compared to their wild-type littermates. In contrast to our *in vitro* experiments, administration of nAbs-Aβ to Tg2576 mice did not lead to an increased inflammatory reaction as measured by intracerebral cytokine levels. Instead IL-1β, TNF-α, IL-6 and IFN-γ levels were unchanged following the administration of nAbs-Aβ to Tg2576 mice. Although the brain is protected by the blood–brain barrier, it has already been shown that nAbs-Aβ can cross it [31]. *In vivo* studies provide an environment with astrocytes, neurons and microglial cells whereas our *in vitro* experiments were performed using microglial cells only. Von Bernhardi *et al*. observed that microglia exposed to Aβ respond with reactive morphological changes, induction of inducible nitric oxide synthase (iNOS), elevated nitric oxide production and decreased reductive metabolism. All these responses were attenuated by the presence of astrocytes [32]. Astrocytes are also involved in Aβ degradation and removal and play an important role in maintaining physiological homeostasis in the brain. However, compared to microglial cells they do not need a stimulus for phagocytosis and at the same time phagocytosis cannot be enhanced by large amounts of IVIg as it can be for microglial cells [16]. Further studies with mixed glial cell cultures are needed to investigate this specific issue.

Interestingly, our observations contrast with those of Minami and colleagues, who showed a profound induction of neuroinflammation following the administration of monoclonal antibodies to mice [33]. One explanation might be the use of a different animal model in the aforementioned study compared to the Tg2576 mouse model in our study. More likely, as a passive immunization strategy, nAbs-Aβ may preclude a neuroinflammatory reaction, unlike monoclonal antibodies *in vivo*. As we only treated mice for 24 hours before measuring intracerebral cytokine levels one could argue that longer treatment periods are needed to assess the influence of administering anti-Aβ antibodies on neuroinflammation. A study by Puli *et al*. also showed significant suppression of TNF-α levels after long-term treatment with IVIg in the APP/PS1 mouse model of AD [34]. Nevertheless, long-term studies with repeated administration of nAbs-Aβ and ft are needed to investigate this issue further and to preclude a rise in pro-inflammatory cytokines upon chronic nAbs-Aβ administration in transgenic mice. With respect to the adverse effects of active immunization observed in an early clinical study, treatment with IVIg and/or nAbs-Aβ might prove a safer approach [5]. By applying two different methods we were able to show that phagocytosis of Aβ oligomers by microglial cells doubles when nAbs-Aβ are pre-administered. This effect was not observed with the same amount of flow-through, indicating a highly specific effect of nAbs-Aβ on the uptake of Aβ from microglial cells. This might be attributed to the formation of antigen-antibody complexes between nAbs-Aβ and Aβ oligomers, which are preferentially taken up by microglial cells. Phagocytosis of antigen-antibody complexes is usually mediated via the Fcγ receptor [35]. In the experimental setup that we used, microglial cells of murine origin were exposed to human IgG. Human Fcγ receptor 1 and murine Fcγ receptor 1 share 65 to 75% homology in their extracellular domains and it is known that human Fcγ receptor 1 can bind murine IgG [36]. Unfortunately, nothing is known on the ability of murine Fcγ receptor 1 to bind to human IgG. As our nAbs-Aβ preparation contains all IgG subclasses, it might be worth testing them with regard to their influence on cytokine secretion and phagocytosis separately in the future. Very recently Smith *et al*. developed a mouse model where all murine Fcγ receptors have been replaced by human Fcγ receptors [37]. It would be ideal to test our human nAbs-Aβ on this mouse model to verify our results and to exclude the impact of species on the immunoglobulin-Fc receptor interaction. However, the positive effect of nAbs-Aβ on Aβ phagocytosis is consistent with results obtained for monoclonal antibodies [24]. Since the amyloid hypothesis postulates that an increase in amyloid-β leads to neurodegeneration, nAbs-Aβ support Aβ oligomer clearance. To determine whether the changes observed for microglial cells have beneficial effects on neurons, we treated primary neurons with conditioned media from microglial cell cultures. Interestingly, we were able to observe a significant improvement in neuron viability from Aβ-treated microglial cells compared to those with co-administered nAbs-Aβ. This was not the case following co-administration of flow-through. We can exclude that this effect is due to a lower amount of Aβ in nAbs-Aβ-treated cell supernatants, even though we observed an increase in Aβ uptake. It is possible that in the co-treated supernatants Aβ is bound to nAbs-Aβ or even modulated in its aggregation state and is therefore less toxic to the neuronal cells. Another explanation could be that the activated microglial cells secrete neurotrophic factors, as it has been reported for lipopolysaccharide (LPS)-treated microglial cells by Nakajima *et al*. [38]. It is thus likely that simultaneous stimulation of Aβ and nAbs-Aβ activates the same signaling pathways in microglial cells that lead to the production of neurotrophic factors. We measured one neurotrophic factor (brain-derived neurotrophic factor (BDNF); data not shown) but were unable to show a significant increase. Other factors such as glial cell line-derived neurotrophic factor (GDNF) may play a role in the observed effect, and further studies are currently underway.

## Conclusions

In summary, we investigated the effects of nAbs-Aβ isolated from IVIg on microglial cells. Although we were not able to rescue microglial cells upon stimulation with Aβ oligomers we showed that nAbs-Aβ have positive effects on the phagocytotic ability of Aβ oligomers. The beneficial effect of microglial-conditioned media on primary neurons is promising, and should be the focus of further research. Our present data demonstrate that nAbs-Aβ are able to enhance Aβ degradation and support neuronal survival without inducing a cerebral inflammatory reaction as shown by unchanged pro-inflammatory cytokine levels *in vivo*. These findings support nAbs-Aβ as a potential therapy to ameliorate Aβ-induced neuronal toxicity in AD.

## Competing interests

This work was partially financed by the Alzheimer Forschungsinitiative (AFI) awarded to JPB.

## Authors’ contributions

MG and DM carried out the laboratory experiments. MG, DM and JPB analyzed the data, interpreted the results and wrote the paper. SR designed and carried out the animal experiments. RD discussed analyses, interpretation and presentation. In addition, he participated in the drafting of the paper. All authors have contributed to, read and approved the final manuscript.
